# Supplementary material for: Modeling glioblastoma heterogeneity as a dynamic network of cell states
Source: Mol Syst Biol. 2021 Sep 16;17(9):e10105. doi: 10.15252/msb.202010105 (PMC8444284; doi:10.15252/msb.202010105)
Supplement: Supplementary file 6 — Source Data for Figure 5 [file MSB-17-e10105-s004.zip › Figure5A_sourcedata/GSEA_3017/nosigdb_stateC.GseaPreranked.1622795002785/RICHARDS_NOWAKOWSKI_SCIENCE_2017_IPC_DIV2_UPREG.html]

Details for gene set RICHARDS\_NOWAKOWSKI\_SCIENCE\_2017\_IPC\_DIV2\_UPREG[GSEA]

|  || Dataset | state13017 |
| Phenotype | NoPhenotypeAvailable |
| Upregulated in class | na\_pos |
| GeneSet | RICHARDS\_NOWAKOWSKI\_SCIENCE\_2017\_IPC\_DIV2\_UPREG |
| Enrichment Score (ES) | 0.7527919 |
| Normalized Enrichment Score (NES) | 6.8547397 |
| Nominal p-value | 0.0 |
| FDR q-value | 0.0 |
| FWER p-Value | 0.0 |
Table: GSEA Results Summary

  

Fig 1: Enrichment plot: RICHARDS\_NOWAKOWSKI\_SCIENCE\_2017\_IPC\_DIV2\_UPREG      
 Profile of the Running ES Score & Positions of GeneSet Members on the Rank Ordered List

  

| PROBE | GENE SYMBOL | GENE\_TITLE | RANK IN GENE LIST | RANK METRIC SCORE | RUNNING ES | CORE ENRICHMENT || 1 | SYNE2 |  |  | 0 | 0.839 | 0.0162 | Yes |
| 2 | TFDP2 |  |  | 1 | 0.787 | 0.0313 | Yes |
| 3 | HES6 |  |  | 5 | 0.694 | 0.0382 | Yes |
| 4 | MAGI1 |  |  | 6 | 0.652 | 0.0507 | Yes |
| 5 | ATAD2 |  |  | 7 | 0.649 | 0.0632 | Yes |
| 6 | FAM111B |  |  | 8 | 0.616 | 0.0751 | Yes |
| 7 | HIST1H4C |  |  | 9 | 0.603 | 0.0867 | Yes |
| 8 | CENPU |  |  | 10 | 0.588 | 0.0980 | Yes |
| 9 | HIST1H1C |  |  | 11 | 0.583 | 0.1093 | Yes |
| 10 | HIST1H1D |  |  | 12 | 0.583 | 0.1205 | Yes |
| 11 | FAM111A |  |  | 13 | 0.576 | 0.1316 | Yes |
| 12 | EZH2 |  |  | 14 | 0.574 | 0.1426 | Yes |
| 13 | CCNE2 |  |  | 15 | 0.570 | 0.1536 | Yes |
| 14 | CLSPN |  |  | 17 | 0.561 | 0.1622 | Yes |
| 15 | C21orf58 |  |  | 18 | 0.559 | 0.1730 | Yes |
| 16 | RRM2 |  |  | 19 | 0.542 | 0.1834 | Yes |
| 17 | HMGB2 |  |  | 20 | 0.534 | 0.1937 | Yes |
| 18 | HIP1 |  |  | 22 | 0.523 | 0.2016 | Yes |
| 19 | CDK6 |  |  | 24 | 0.518 | 0.2094 | Yes |
| 20 | XRCC2 |  |  | 26 | 0.495 | 0.2168 | Yes |
| 21 | CHAF1A |  |  | 27 | 0.494 | 0.2263 | Yes |
| 22 | UBE2T |  |  | 31 | 0.475 | 0.2289 | Yes |
| 23 | HIST1H1B |  |  | 35 | 0.460 | 0.2313 | Yes |
| 24 | SAMD1 |  |  | 36 | 0.457 | 0.2401 | Yes |
| 25 | CDCA7L |  |  | 37 | 0.453 | 0.2488 | Yes |
| 26 | GMNN |  |  | 38 | 0.453 | 0.2576 | Yes |
| 27 | ORC6 |  |  | 39 | 0.450 | 0.2662 | Yes |
| 28 | MMS22L |  |  | 40 | 0.448 | 0.2749 | Yes |
| 29 | ESCO2 |  |  | 43 | 0.440 | 0.2790 | Yes |
| 30 | UHRF1 |  |  | 45 | 0.436 | 0.2852 | Yes |
| 31 | RAD51AP1 |  |  | 46 | 0.435 | 0.2936 | Yes |
| 32 | PCNA |  |  | 48 | 0.435 | 0.2998 | Yes |
| 33 | TMPO |  |  | 49 | 0.432 | 0.3082 | Yes |
| 34 | DTL |  |  | 50 | 0.430 | 0.3164 | Yes |
| 35 | HIST1H1E |  |  | 51 | 0.430 | 0.3247 | Yes |
| 36 | TOP2A |  |  | 52 | 0.426 | 0.3329 | Yes |
| 37 | SPC25 |  |  | 53 | 0.415 | 0.3409 | Yes |
| 38 | BRCA1 |  |  | 54 | 0.410 | 0.3488 | Yes |
| 39 | DHFR |  |  | 55 | 0.405 | 0.3566 | Yes |
| 40 | WDR76 |  |  | 56 | 0.403 | 0.3644 | Yes |
| 41 | TYMS |  |  | 60 | 0.390 | 0.3654 | Yes |
| 42 | PHIP |  |  | 61 | 0.387 | 0.3729 | Yes |
| 43 | RRM1 |  |  | 62 | 0.386 | 0.3803 | Yes |
| 44 | SMC2 |  |  | 63 | 0.386 | 0.3877 | Yes |
| 45 | E2F7 |  |  | 69 | 0.377 | 0.3842 | Yes |
| 46 | CSRP2 |  |  | 72 | 0.376 | 0.3871 | Yes |
| 47 | DEK |  |  | 74 | 0.373 | 0.3921 | Yes |
| 48 | NASP |  |  | 75 | 0.371 | 0.3992 | Yes |
| 49 | POLD3 |  |  | 76 | 0.367 | 0.4063 | Yes |
| 50 | CENPH |  |  | 77 | 0.366 | 0.4134 | Yes |
| 51 | SMC4 |  |  | 78 | 0.365 | 0.4204 | Yes |
| 52 | ITGB3BP |  |  | 79 | 0.364 | 0.4274 | Yes |
| 53 | CDCA5 |  |  | 81 | 0.361 | 0.4322 | Yes |
| 54 | GINS2 |  |  | 83 | 0.360 | 0.4370 | Yes |
| 55 | BARD1 |  |  | 86 | 0.357 | 0.4395 | Yes |
| 56 | DUT |  |  | 87 | 0.356 | 0.4464 | Yes |
| 57 | MIS18BP1 |  |  | 89 | 0.355 | 0.4510 | Yes |
| 58 | CARHSP1 |  |  | 90 | 0.353 | 0.4578 | Yes |
| 59 | MCM7 |  |  | 91 | 0.352 | 0.4646 | Yes |
| 60 | USP1 |  |  | 92 | 0.352 | 0.4714 | Yes |
| 61 | ASRGL1 |  |  | 93 | 0.350 | 0.4781 | Yes |
| 62 | ATAD5 |  |  | 97 | 0.345 | 0.4783 | Yes |
| 63 | FEN1 |  |  | 98 | 0.342 | 0.4849 | Yes |
| 64 | NUSAP1 |  |  | 100 | 0.340 | 0.4892 | Yes |
| 65 | CKB |  |  | 102 | 0.335 | 0.4935 | Yes |
| 66 | NCAPD3 |  |  | 103 | 0.334 | 0.4999 | Yes |
| 67 | MCM8 |  |  | 104 | 0.331 | 0.5063 | Yes |
| 68 | SLBP |  |  | 106 | 0.331 | 0.5105 | Yes |
| 69 | ZNF43 |  |  | 107 | 0.330 | 0.5169 | Yes |
| 70 | INSM1 |  |  | 108 | 0.329 | 0.5232 | Yes |
| 71 | MCM3 |  |  | 114 | 0.324 | 0.5187 | Yes |
| 72 | H2AFX |  |  | 115 | 0.322 | 0.5248 | Yes |
| 73 | HIRIP3 |  |  | 116 | 0.321 | 0.5310 | Yes |
| 74 | UBTF |  |  | 117 | 0.320 | 0.5372 | Yes |
| 75 | PSMC3IP |  |  | 118 | 0.319 | 0.5433 | Yes |
| 76 | TIMELESS |  |  | 119 | 0.319 | 0.5495 | Yes |
| 77 | CCDC34 |  |  | 120 | 0.318 | 0.5556 | Yes |
| 78 | HAT1 |  |  | 121 | 0.318 | 0.5617 | Yes |
| 79 | RHOBTB3 |  |  | 124 | 0.313 | 0.5634 | Yes |
| 80 | SMC1A |  |  | 125 | 0.312 | 0.5695 | Yes |
| 81 | HMGN2 |  |  | 126 | 0.312 | 0.5755 | Yes |
| 82 | DNMT1 |  |  | 127 | 0.311 | 0.5815 | Yes |
| 83 | FBXO5 |  |  | 130 | 0.308 | 0.5830 | Yes |
| 84 | SSX2IP |  |  | 131 | 0.307 | 0.5890 | Yes |
| 85 | H3F3A |  |  | 132 | 0.304 | 0.5948 | Yes |
| 86 | KIF15 |  |  | 136 | 0.302 | 0.5941 | Yes |
| 87 | NCAPG |  |  | 137 | 0.301 | 0.5999 | Yes |
| 88 | RFC4 |  |  | 138 | 0.300 | 0.6057 | Yes |
| 89 | CENPK |  |  | 139 | 0.299 | 0.6115 | Yes |
| 90 | LMNB1 |  |  | 142 | 0.293 | 0.6128 | Yes |
| 91 | ASF1B |  |  | 143 | 0.291 | 0.6184 | Yes |
| 92 | HNRNPAB |  |  | 144 | 0.290 | 0.6240 | Yes |
| 93 | ASCL1 |  |  | 145 | 0.290 | 0.6296 | Yes |
| 94 | C12orf57 |  |  | 146 | 0.289 | 0.6351 | Yes |
| 95 | CCND2 |  |  | 149 | 0.288 | 0.6363 | Yes |
| 96 | SIVA1 |  |  | 151 | 0.286 | 0.6397 | Yes |
| 97 | SUPT16H |  |  | 153 | 0.286 | 0.6430 | Yes |
| 98 | RFC3 |  |  | 156 | 0.283 | 0.6441 | Yes |
| 99 | RNASEH2B |  |  | 158 | 0.282 | 0.6474 | Yes |
| 100 | CEP78 |  |  | 159 | 0.281 | 0.6528 | Yes |
| 101 | ZNF93 |  |  | 160 | 0.281 | 0.6582 | Yes |
| 102 | CBFA2T2 |  |  | 162 | 0.280 | 0.6615 | Yes |
| 103 | PARP1 |  |  | 163 | 0.280 | 0.6669 | Yes |
| 104 | MYBL2 |  |  | 164 | 0.278 | 0.6722 | Yes |
| 105 | H2AFY |  |  | 165 | 0.278 | 0.6776 | Yes |
| 106 | CHD7 |  |  | 166 | 0.277 | 0.6829 | Yes |
| 107 | VRK1 |  |  | 169 | 0.275 | 0.6839 | Yes |
| 108 | HELLS |  |  | 171 | 0.271 | 0.6869 | Yes |
| 109 | C19orf48 |  |  | 173 | 0.271 | 0.6900 | Yes |
| 110 | MAD2L1 |  |  | 174 | 0.270 | 0.6952 | Yes |
| 111 | NKAIN3 |  |  | 175 | 0.268 | 0.7003 | Yes |
| 112 | RBBP8 |  |  | 177 | 0.267 | 0.7033 | Yes |
| 113 | RIF1 |  |  | 178 | 0.266 | 0.7084 | Yes |
| 114 | POLA1 |  |  | 179 | 0.266 | 0.7135 | Yes |
| 115 | MSH2 |  |  | 180 | 0.265 | 0.7187 | Yes |
| 116 | DDX11 |  |  | 181 | 0.265 | 0.7237 | Yes |
| 117 | SNCAIP |  |  | 182 | 0.264 | 0.7288 | Yes |
| 118 | RMI1 |  |  | 183 | 0.263 | 0.7339 | Yes |
| 119 | NCAPG2 |  |  | 187 | 0.260 | 0.7324 | Yes |
| 120 | XRCC5 |  |  | 188 | 0.259 | 0.7374 | Yes |
| 121 | MCM4 |  |  | 189 | 0.259 | 0.7424 | Yes |
| 122 | BAZ2B |  |  | 190 | 0.259 | 0.7474 | Yes |
| 123 | CDK1 |  |  | 191 | 0.255 | 0.7523 | Yes |
| 124 | E2F3 |  |  | 194 | 0.252 | 0.7528 | Yes |
| 125 | PGAM1 |  |  | 218 | -0.258 | 0.7080 | No |
| 126 | FAM200B |  |  | 235 | -0.260 | 0.6784 | No |
| 127 | CLIC1 |  |  | 237 | -0.261 | 0.6812 | No |
| 128 | PHLDA1 |  |  | 295 | -0.274 | 0.5631 | No |
| 129 | CREB5 |  |  | 309 | -0.281 | 0.5404 | No |
| 130 | TMEM158 |  |  | 315 | -0.284 | 0.5351 | No |
| 131 | GNB4 |  |  | 335 | -0.289 | 0.4995 | No |
| 132 | RPS24 |  |  | 417 | -0.333 | 0.3306 | No |
| 133 | TGIF1 |  |  | 502 | -0.421 | 0.1569 | No |
| 134 | CA12 |  |  | 586 | -0.717 | -0.0090 | No |
| 135 | FABP5 |  |  | 596 | -1.477 | 0.0000 | No |
Table: GSEA details [plain text format]

  

Fig 2: RICHARDS\_NOWAKOWSKI\_SCIENCE\_2017\_IPC\_DIV2\_UPREG: Random ES distribution      
 Gene set null distribution of ES for **RICHARDS\_NOWAKOWSKI\_SCIENCE\_2017\_IPC\_DIV2\_UPREG**
